# Clinical Value of NOTCH1 Mutations Detection among Chronic Lymphocytic Leukemia Patients

**DOI:** 10.31557/APJCP.2020.21.5.1295

**Published:** 2020-05

**Authors:** Salah Are, Mohamed El-Agdar, Osama Salama, Tarek Abouzeid, Mohamed Sabry

**Affiliations:** 1 *Hematology Unit, Clinical Pathology Department, Mansoura Faculty of Medicine, Mansoura University, Egypt. *; 2 *Hematology Unit, Internal Medicine Department, Mansoura University Oncology Center, Mansoura Faculty of Medicine, Mansoura, Egypt.* ^*.*^

**Keywords:** CLL, NOTCH1, mutations, prognosis

## Abstract

**Background:**

The data about the clinical impact of NOTCH1 mutations among Egyptians B – cell chronic lymphocytic patients is not previously identified. We herein, evaluate the prevalence and the prognostic significance of neurogenic locus notch homolog protein-1 (NOTCH1) mutations in B- cell lymphocytic leukemia (B-CLL).

**Methods:**

A cohort of 105 Egyptian B-CLL patients aging from 43 to 86 years. PCR products including NOTCH1 exon 26, 27, and distal part of exon 34 expanding the sequences encoding transcription activation domain (TAD) and a peptide sequence rich in proline (P), glutamic acid (E), serine (S), threonine (T) (PEST domains) were sequenced by direct DNA Sanger sequencing.

**Results:**

NOTCH1 mutations were detected in 48/105 of patients (45.7%). Mutations in B-CLL patients are insertions (n=21), point mutations (n=18) and deletions (n=12). NOTCH1 mutations showed significant impact on prognosis of B-CLL patients as they were associated with increased bone marrow lymphocytes, more relapse and high incidence of mortality, shortened overall survival and progression free survival, and lymphocytes doubling time, when compared with NOTCH1 wild type B-CLL patients (P= 0.001; 0,005; 0.042; 0.049; 0.008; 0.049 respectively).

**Conclusion:**

NOTCH1 mutations were considered as bad prognostic marker in B-CLL and suggested to be included in risk stratification of B-CLL patients at diagnosis.

## Introduction

CLL is considered the most common type of leukemia in Western countries. It represents 40% of leukemia in adult patients. On the other hand, African, Asian and Caribbean people have lower CLL frequency (Fabbri et al., 2016). Multiple studies revealed that NOTCH1 plays a prominent role in T-cell acute lymphoblastic leukemia, breast cancer, choriocarcinoma (Leong et al., 2006; Pang et al., 2010), and CLL (Mansouri et al., 2013). 

NOTCH1 is a class I transmembrane (TM) protein which directly transduce extracellular signal into gene expression changes which function as ligand activated transcription factors. Interaction of the receptor with D like and Jagged ligands expressed on the surface of neighboring cells initiate NOTCH1 signal. This ligand receptor interaction cleaves extracellular domains of the NOTCH1 receptor by the metalloprotease a disintegrin and metalloproteinase domain-containing protein 10 (ADAM10), that facilitates subsequent proteolytic cleavage by g secretase complex in the transmembrane region of the receptor (Martin et al., 2017).

NOTCH1 pathway is an evolutionarily conserved signaling pathway that regulates cell fate specification differentiation, and patterning. Mutations of NOTCH ligands and receptors are implicated in numerous congenital and acquired human diseases. NOTCH1 Mutations were found to be a poor prognostic marker in CLL (Mansouri et al., 2013). Also, the prevalence of NOTCH1 mutation was shown to be higher with increased aggressiveness of disease (Rossi et al., 2012; Del Giudice et al., 2012). The data about the prevalence and impact and types of NOTCH1 mutations in CLL among Egyptian patients still no identified.

The aim of this study was to detect the prevalence of NOTCH1 mutations among a cohort of Egyptian patients with B cell CLL, as well as, it’s relation to disease behavior and patient outcome.

## Materials and Methods

*Patients and methods*

This study was carried out on 105 newly diagnosed Patients with B - CLL attending the oncology center of Mansoura University, Egypt; after taken the informed consent from all patients. The study was approved by local ethical committee. The age of B-CLL patients ranged from 43 to 86 years. B-CLL patients were diagnosed by complete blood counts (CBC), blood smear examination, and immunophenotyping by flowcytometry. B- CLL cases were classified according to Binet staging system.

Median follow up of B cell CLL patients was 20 months ranging from 12 months to 31 months. During follow up we recorded remission, overall survival (OS) progression free survival (PFS) and lymphocyte doubling time (LDT) in cases with B- CLL. 

75 of 105 B cell CLL cases received chemotherapy. Protocols used were composed of chlorambucil, and COP protocol (cyclophosphamide, vincristine, prednisolone) or CHOP (cyclophosphamide, vincristine, doxorubicin, prednisone) when there is progression of the disease (Marcus et al., 2005; Bo et al., 2014). 

*Follow up and prognostic criteria*

Complete remission occurs when blood lymphocytes less than 4,000/μl, hemoglobin more than 11g/dL, platelets count more than 100,000 /μl, neutrophils more than 1,500 /μl, and bone marrow is normocellular with no B lymphoid nodules, with less than 30% lymphocytes with no hepatosplenomegaly, or lymphadenopathy (Hallek et al., 2008).

Partial remission occurs when blood lymphocytes reduced ≥ 50% from baseline, hemoglobin more than 11 g/dL or increase ≥ 50% over baseline, neutrophils more than 1500/μl or ≥ 50% improvement over baseline, platelet counts more than 100 000/μl or increase ≥ 50% over baseline, with 50% reduction in B-lymphoid nodules or marrow infiltration or with decrease ≥ 50% in hepatosplenomegaly, and lymphadenopathy (Hallek et al., 2008).

Disease progression occurs when blood lymphocytes increased ≥ 50% from baseline, decrease of hemoglobin more than 2 g/dL from baseline secondary to CLL platelet counts decreased ≥ 50% from baseline, with increase ≥ 50% in hepatosplenomegaly and lymphadenopathy (Hallek et al., 2008).

Stable disease is the case who not achieved either partial remission (PR) or complete remission (CR) and didn’t show disease progression (Hallek et al., 2008). All responses rather than CR and PR are considered as treatment failure. Refractory disease includes treatment failure (as previously described) or patient exhibited progression within 6 months from the last chemotherapy. Relapse is considered when patient who has previously achieved the above criteria of a CR or PR, but after 6 or more months, show evidence of disease progression (Hallek et al., 2008). 

Lymphocyte doubling time (LDT) is the time needed for doubling of the count of lymphocytes in relation to its count at diagnosis (Molica and Alberti 1987).

Progression free survival (PFS) is the time from study entry until evidence of disease progression or death. Overall survival (OS) is the time from study entry until death from any cause or last contact with patient (Hallek et al., 2008).

*Methods*

Three milliliters of peripheral blood were taken on ethylenediaminetetraacetic (EDTA) tube for CBC, flowcytometry and DNA extraction (QIAGEN DNA Extraction Kit Protocol). Peripheral blood films were prepared for morphological examination. Bone marrow aspirate was done in some cases for morphological examination and detection of bone marrow infiltration. DNA amplification was performed by conventional PCR with NOTCH1 mutation analysis by direct Sanger sequencing of PCR products encompassing NOTCH1 exon 26, 27, and the distal part of exon 34 including the sequences encoding the TAD and PEST domain because these parts are the hot areas for presence of mutations. Sequences of Primer were as follows: Exon 26 Forward (FW): 5-GGAAGGCGGCCTGAGCGTGTC-3; Exon 26 RV: 5-ATTGACCGTGGGCGCCGGGTC-3; with annealing temperature of 67.5, C Exon 27 FW: 5-GCCTCAGTGTCCTGCGGC-3; Exon 27 Reverse (RV): 5-GCACAAACAGCCAGCGTGTC-3; with annealing temperature of 60 C, Exon 34 FW1(TAD): 5-GCTGGCCTTTGAGACTGGC-3; Exon 34 RV1(TAD): 5-GCTGAGCTCACGCCAAGGT-3; with annealing temperature of 63 C, Exon 34 FW2 (Pest): 5-CAGATGCAGCAGCAGAACCTG-3; and exon 34 RV2(Pest): 5-AAAGGAAGCCGGGGTCTCGT-3 with annealing temperature of 64 C.

*Statistical Analysis*

The statistical analysis of data was done using excel program (Microsoft Office 2013) and SPSS (statistical package for social science) program (SPSS, Inc, Chicago, IL) version 20. Qualitative data were presented as frequency and percentage. Chi square and Fisher’s exact tests were used to compare groups. Quantitative data were presented by mean, SD or median and range. Comparisons between two groups were done using t-test or Man Whitney (for non-parametric). Kaplan–Meier test was used for survival analysis and the statistical significance of differences among curves was determined by Log-Rank test. Cox regression analysis was used for prediction of OS. P-value less than 0.05 was considered statistically significant.

## Results

*Mutational Analysis*

**Figure 1:**
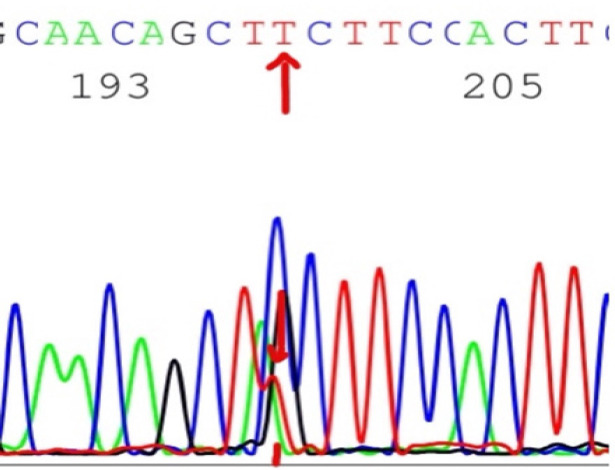
The Point Mutation C4766T in HD Domain (exon 26) of *NOTCH1* Gene with Replacement of Cytosine with Thiamine

The frequency of NOTCH1 mutations were not significantly associated with the age or sex (P>0.05 for both) (Table 1). NOTCH1 mutations were detected in 48 out of 105 of B - CLL patients (45.7%). The highest frequency of the detected mutations was in exon 34 (Pest domain) in 30 cases out of 105 cases (28.5%), followed by exon 26 in 12 out of 105 cases (11.4%), followed by TAD domain of exon 34 in 3 out of 105 cases (2.8%)) and exon 27 in 3 out of 105 cases (2.8%). Only 3 case had double mutations. Twenty-one of NOTCH1 mutations are insertions and 18 are point mutations (Figure 1) and 12 are deletions (Table 2). No significant association of NOTCH1 mutations with the age or sex (P>0.05 for both) (Table 2). 

*Hematological analysis*

**Figure 2:**
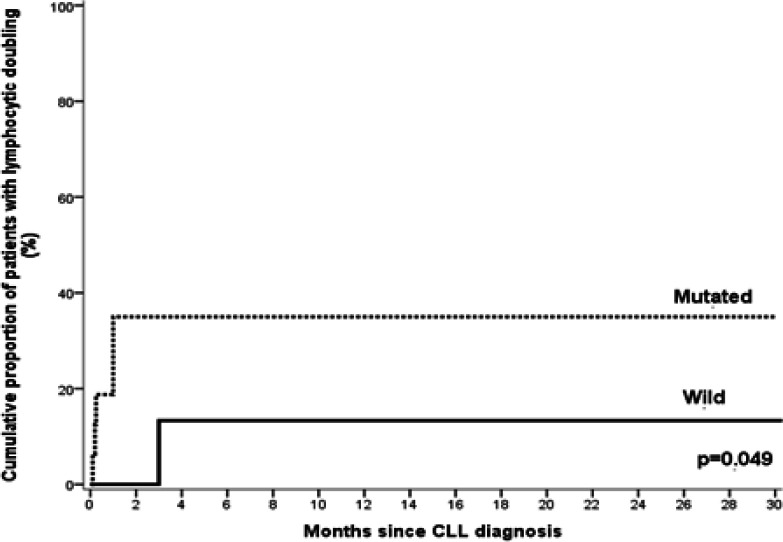
Lymphocyte Doubling Time Hazard Curve in Relation to NOTCH1 Mutations in CLL Patients. NOTCH1 mutations were significantly associated with shorter mean lymphocyte doubling time than that associated with wild type (P= 0.049).

NOTCH1 mutations were significantly associated with higher BM lymphocytes in B cell CLL patients (P=0.001). However, regarding other hematological parameters there is no significant differences between B cell CLL patients with NOTCH1 mutations and those without (P>0.05 for all) (Table 3). On the other hand, NOTCH1 mutations were significantly associated with shorter mean LDT as compared to B cell CLL patients with wild type (P = 0.049) (Figure 2). 

*Clinical impact of NOTCH1 mutations*

Cox regression analysis was conducted for prediction of LDT within B cell CLL patients, using age, gender, ALC, BM lymphocytes, staging, CD38, and NOTCH1 mutations as covariates. NOTCH1 mutations and ALC were found to be risk factors for shorter LDT in both uni and multivariate analyses (Table 4).

There is significant association between NOTCH1 mutations and relapse, disease progression, and mortality when compared to B cell CLL patients with wild type. Cases who relapsed in B cell CLL cases were 0 % in wild type cases and 37.5 % in mutated cases (P=0.005). Progression was observed in 10.5 % of wild type cases and in 50 % of mutated cases (P=0.022). Mortality rate was higher in mutated cases (37.5%) as compared to those with wild type (5.3%) (P=0.042). Other clinical parameters in B cell CLL case had no differences in relation to NOTCH1 mutation (Table 5). 

**Figure 3.**
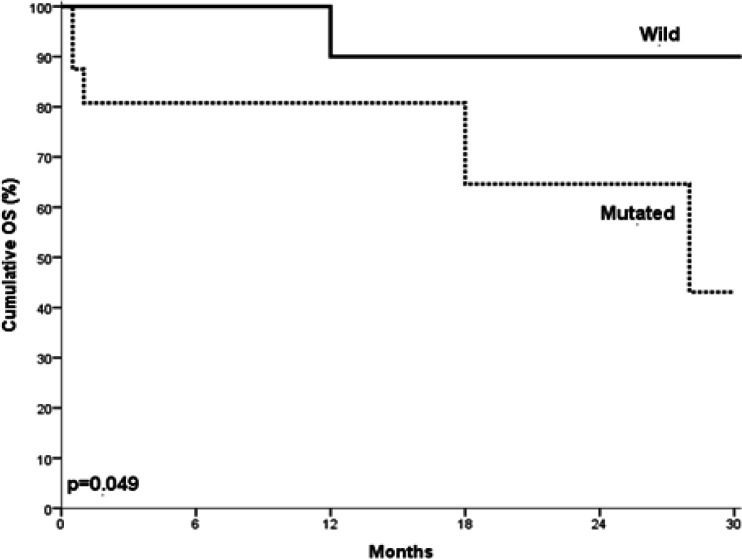
Overall Survival in Relation to NOTCH1 Mutations in Studied B cell CLL Cases. CLL cases with NOTCH1 mutations showed statistically significantly shorter overall survival time in comparison with those with no mutations (P= 0.049).

**Figure 4:**
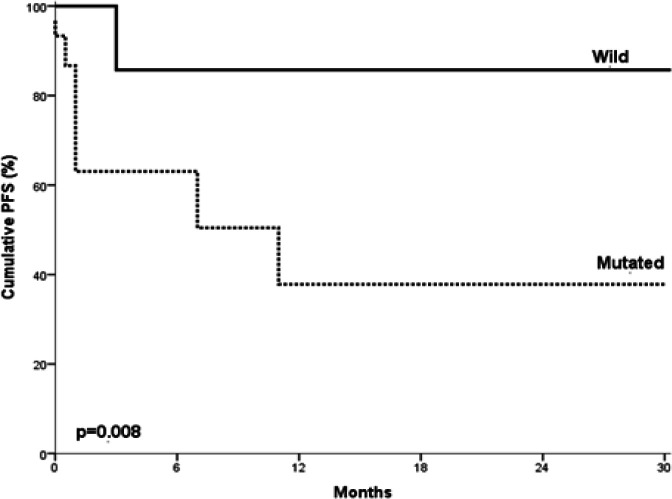
Progression Free Survival in Relation to NOTCH1 Mutations in Studied B cell CLL Cases. CLL cases with NOTCH1 mutations showed significantly lower progression free survival rates, shorter progression free survival times in comparison to those with no mutations (P= 0.008).

NOTCH1 mutated B cell CLL cases showed statistically significant shorter OS when compared to those with no mutations (P= 0.049) (Figure 3). Moreover, the mutated B cell CLL cases showed statistically significant shorter PFS when compared to those with no mutations (P= 0.008) (Figure 4). 

Cox regression analysis was conducted for prediction of OS within studied B cell CLL cases, using age, gender, BM lymphocytes, ALC, CD38, staging, NOTCH1 mutations as covariates. NOTCH1 mutations were considered as independent risk factor for shorter OS (Table 6). Cox regression analysis was conducted for prediction of PFS in B cell CLL patients, using age, gender, ALC, BM lymphocytes, staging, CD38, NOTCH1 mutations as covariates. NOTCH1 mutation was found to be a bad prognostic factor for shortening of PFS (Table 7). 

**Table 1:** Comparison between NOTCH1 Mutated and Wild Type B-CLL Cases in Relation to Age and Gender

		Wild type NOTCH1		Mutated NOTCH1		P value
CLL		N=57		N=48		
Age (years)	Mean / SD	62.7	14.4	64.1	9.3	0.753
Male	N / %	45	78.9	39	81.25	0.865
Female	N / %	12	21.1	9	18.75	

**Table 2:** Types of NOTCH1 Mutations in All Studied CLL Cases

Case No	domain	Type of mutation	Nucleotide change	Amino acid change
1;50;51;55	HD-N exon 26	Point mutation	c.*4766C>T	S1590F
3;60;70	Pest exon 34	Deletion	c.7486 del A	N2497T
4;20;22	Pest exon 34	Insertion	c. 7526_7527 ins C	E2516L H2508A
5;8;65	Pest exon 34	Point mutation	c.*550C>T	P2518L
6;74;72	Pest exon 34	Insertion	c. 7542_7483 insT	S2517F
10;23;27	Pest exon 34	Insertion	c. 7542_7483 insG	E2516G S2517V
11;54;61	Tad exon 34	Deletion	c.6821 del C	T2285A
15;44;71	HD-N exon 26	Point mutation	c.*4916A>C	E1636A
16;90;92	HD-N exon 26	Deletion	4609 Del A	C1537L K1538Q
18;73;104	HD-N exon 26	Point mutation	c.*5011G>A	V1672I
20;61;69	HD-C exon 27	Point mutation	c.*5027T>A	V1677D
25;44;77	Pest exon 34	Insertion	c. 7543_7544 ins A	S2517v
28, 30;66	Pest exon 34	Insertion	c. 7481_7482 insG	DEL DNT2496 INS GQH
29;57;88	Pest exon 34	Deletion	c.7486 del A	N2497T T2498P
		Insertion	c. 7489_7490 insC	
33;101	Pest exon 34	Point mutation	c. *7513C>G	Del peh2506 ins AGA

Del, deletion; ins, insertion

**Table 3:** Comparison between NOTCH1 Mutated and Wild Type B-CLL Cases in Relation to Hematological Data

CLL	Wild type NOTCH1 N=57			Mutated NOTCH1 N=48			*P*-value
	Median	Range		Median	Range		
Total leucocytic count (X10^9^/L)	63.6	6.0	175	38.5	16.8	165.2	>0.05
Hemoglobin concentration (g/dL)	12.9	7.0	15	11.5	7.0	14.0	>0.05
Platelets count (X10^9^/L)	164.0	42.0	267	161.5	64.0	573	>0.05
Absolute lymphocytic count (X10^9^/L)	28.5	5.2	153	30.3	10.1	137.7	>0.05
BM lymphocytes (%)	58.0	38.0	85	80.0	40.0	96.0	0.001

**Table 4:** Cox Regression Analysis for Prediction of Lymphocyte Doubling Time in B-CLL Cases

	Univariate				Multivariate			
	*P*-value	HR	95% CI		*P*-value	HR	95% CI	
Age (years)	>0.05	1.032	0.961	1.108				
Male	>0.05	2.993	0.215	14.222				
BM lymphocytes(X10^9^/L)	0.097	1.05	0.991	1.112				
Absolute lymphocyte count(X10^9^/L)	0.046	1.313	1.023	1.927	0.046	1.015	1.001	1.03
Positive CD38	>0.05	1.711	0.383	7.657				
Advanced stage	>0.05	1.364	0.305	6.1				
NOTCH1 mutation	0.029	4.399	1.842	22.99	0.044	4.94	1.914	26.699

**Table 5:** Comparison between NOTCH1 Mutated and Wild Type B-CLL Cases in Relation to Clinical Outcome

Clinical outcome	Wild type NOTCH1 N=57		Mutated NOTCH1 N=48		*P* value
	No.	%	No.	%	
Complete remission	21	36.8	6	12.5	0.001
Partial remission	15	26.3	21	43.8	>0.05
Refractory	21	36.8	21	43.8	>0.05
Progression	6	10.5	18	50	0.001
Relapse	0	0.0	18	37.5	0.001
Mortality rate	3	5.3	15	31.3	0.001

CR, complete remission; PR, partial remission

**Table 6:** Cox Regression Analysis for Prediction of Overall Survival in B-CLL Cases

	*P*-value	HR	95% CI	
Age (years)	0.078	1.086	0.991	1.192
Male	0.559	2.392	0.012	9.61
BM lymphocytes (X10^9^/L)	0.447	1.023	0.965	1.085
ALC (X10^9^/L)	0.136	1.012	0.996	1.027
Positive CD38	0.194	3.098	0.563	17.039
Advanced stage	0.409	1.980	0.392	10.002
NOTCH1 mutation	0.045	2.286	1.727	15.361

ALC, Absolute lymphocyte counts

**Table 7:** Cox Regression Analysis for Prediction of Progression Free Survival in CLL Cases

	P	HR	95% CI	
Age (years)	0.975	0.999	0.928	1.075
Male	0.436	2.288	0.285	18.354
BM lymphocytes(X10^9^/L)	0.079	1.05	0.994	1.109
ALC(X10^9^/L)	0.098	1.011	0.998	1.023
Positive CD38	0.664	1.342	0.356	5.059
Advanced stage	0.423	1.714	0.459	6.403
NOTCH1 mutation	0.022	6.343	1.308	30.768

HR, high risk

## Discussion

NOTCH1 mutations were detected in 45.7% of B cell CLL cases; and this figure is higher than was previously reported 24% (Van Vlierberghe et al., 2013). This may be due to difference in race and in clinical conditions of our patients which may have more progressive disease due to the suggested difference in the molecular and biological behaviors. Also, NOTCH1 mutations is involved in pathogenesis and determination of outcome of CLL patients (Gianfelici et al., 2012; Van Vlierberghe et al., 2013).

No statistically significant differences in age and gender in relation to NOTCH1 mutations in B cell CLL cases. This was in agreement the data reported by Bo et al., (2014) and Villamor et al., (2013). CLL cases with NOTCH1 mutations had significantly higher bone marrow (BM) lymphocytes as compared to those with wild type. No other significant variations in the hematologic data in the relation to NOTCH1 mutation were detected. Except for BM lymphocytes this data is consistent with that reported by Villamor et al., (2013). NOTCH1 mutations were significantly associated with shorter mean LDT than that associated with wild type; a finding consistent with the data reported by Villamor et al., (2013). This may be due to the effect of NOTCH1 mutations which lead to truncation of the C-terminal PEST domain with imperfect degradation of the intracellular portion of NOTCH1 (ICN1) receptor with increased NOTCH1 signaling that leads to longer cell survival and decreased apoptosis (Villamor et al., 2013). 

NOTCH1 mutated B cell CLL patients were classified in higher Binet staging when compared with wild type patients; but this did not reach the statistically significant level. This was in agreement with the data reported by Del Giudice et al., (2012) and Villamor et al., (2013). In the current study there was no significant differences between CLL patients with NOTCH1 mutations and those with wild type as regards the response to induction chemotherapy. In contrast; Bo et al., (2014) reported higher rates of partial remission and complete remission (CR) in cases with wild type NOTCH1.

NOTCH1 mutated B cell CLL patients showed more disease progression as compared with wild type CLL patients. This was in agreement with the findings that reported by Villamor et al., (2013) and Bo et al., (2014). Moreover, NOTCH1 mutated B cell CLL patients showed higher rate of relapse and mortality as compared with wild type CLL patients. This finding was consistent with the data reported by Villamor et al., (2013). NOTCH1 mutated B-CLL cases showed shorter PFS as compared with wild type CLL patients. This data was in agreement with the results reported by Oscier et al., (2013) and Villamor et al., (2013). However; NOTCH1 mutated B cell CLL cases showed longer PFS as compared with wild type CLL cases in fludarabine resistant patients who are on alemtuzumab therapy (Schnaiter et al., 2013). NOTCH1 mutated B cell CLL cases showed shorter overall survival (OS) as compared with NOTCH1 wild type patients. This was consistent with the data reported by Oscier et al., (2013). On the other hand, Schnaiter et al., (2013) reported that there was no statistically significant correlation between NOTCH1 mutation and OS in CLL patient.

B- CLL patients with NOTCH1 mutations in our study were significantly associated with poor prognosis (higher rate of relapse, disease progression, mortality) shortened OS, LDT, PFS as compared to CLL patients with wild type. This was in agreement with the data reported by Weissmann et al., (2013) and Bo et al., (2014). This may be explained on the basis that activation of NOTCH1 signaling leading to longer cell survival and resistance to apoptosis that resulted from NOTCH1 mutations that leads to truncation of the C-terminal PEST domain with decreased degradation intracellular portion of NOTCH1 receptor degradation ICN1 (Zuurbier et al., 2010). The limitation of the current study is the small patient’s number.

In conclusion, NOTCH1 mutations were frequently detected in B cell CLL patients. NOTCH1 mutations had deleterious impact on patient outcome in B- CLL patients. Identification of NOTCH1 mutations in B-CLL patients at diagnosis is recommended for better stratification. Large scale prospective study is recommended to validate our suggestion.
